# Primary intra-abdominal malignant fibrous histiocytoma presenting as pyrexia of unknown origin – report of a case with review of literature

**DOI:** 10.1186/1477-7800-3-15

**Published:** 2006-06-22

**Authors:** NA Qureshi, MT Hallissey, JW Fielding, D Gourevitch

**Affiliations:** 1 Department of upper Gastro-intestinal surgery, Queen Elizabeth Hospital, Edgbaston, Birmingham, UK

## Abstract

Primary intra-abdominal malignant mesenchymal tumours are very rare and there are not many cases of visceral malignant fibrous histiocytoma in the English literature. We report a new case of abdominal malignant fibrous histiocytoma presenting as abdominal pain and pyrexia of unknown origin in a 54 year old female followed by a brief review of literature. Presentation with pyrexia of unknown origin is extremely rare in this condition.

## Background

The term Malignant Fibrous Histiocytoma (MFH) was originally described by O'Brien and Stout in 1964 [1] to cover a variety of pleomorphic soft tissue sarcomas derived from histiocytes capable of fibroblastic transformation. MFH is a common sarcoma of mesenchymal origin affecting soft tissues of the body, especially the extremities and retroperitoneum. Primary intra-abdominal MFH is a rare disease and few cases are reported in the English literature. Its presentation as high-grade pyrexia of unknown origin (PUO) is extremely unusual. We report a case of malignant fibrous histiocytoma affecting colon, spleen, left hemi diaphragm and distal pancreas in a 54 year old female, who presented with abdominal pain and pyrexia of unknown origin.

## Case report

A 54 year old female presented to our surgical outpatient clinic with abdominal pain and pyrexia. Abdominal pain was of recent onset and mainly in the left upper abdomen but she had fever for at least 6 months. Her pyrexia was high grade (39.5–40 C^0^), intermittent, and most common in the early morning. Clinical examination revealed a firm, vaguely defined, tender mass in the left upper quadrant of the abdomen. Blood results showed persistently high ESR (>100), high CRP (>240), leukocytosis, mildly raised Alkaline Phosphatase and anaemia (normochromic, normocytic). There was no overt source of infection that could account for the fever. Repeated blood cultures did not yield any bacterial growth. NM Leukocyte HMPAO scan showed no convincing abnormality to help localise an infective focus. There was no improvement in pyrexia after treating the patient with broad-spectrum antibiotics.

**Figure 1:**
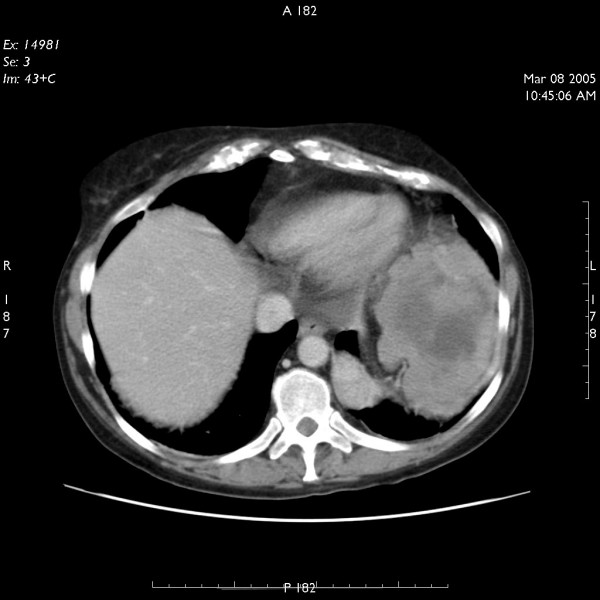
CT scan of abdomen showing extent of primary tumour.

CT scan of the abdomen was performed which showed three heterogeneous masses in left upper abdomen relating to splenic flexure of colon, pancreatic body and tail and left crus of the diaphragm (figure 1). There was evidence of central necrosis in two of these masses. A trucut biopsy of the mass was performed and histology revealed spindle cell leiomyosarcoma.

The patient was consented for surgery and distal pancreatectomy, splenectomy, left adrenalectomy and resection of part of colon was undertaken to remove the tumour via a left thoracoabdominal approach. The patient had an uneventful post-operative recovery and her pyrexia resolved completely following surgery.

Three different specimens were submitted for histology. Specimen A had a smooth external surface, measured 180 × 140 × 130 mm and comprised of a multi-nodular mass with spleen and a loop of large bowel. The cut surface of the tumour was grey with large areas of necrosis up to 80%. The bowel segment appeared to be entrapped and focally infiltrated by the tumour up to the mucosa; however, both cut ends margins were free of tumour. Specimen B comprised of lobulated mass with fatty tissue and measured 140 × 60 × 30 mm. Some adrenal tissue was identified which was not involved by the tumour. Specimen C consisted of four pieces of haemorrhagic tissue.

**Figure 2:**
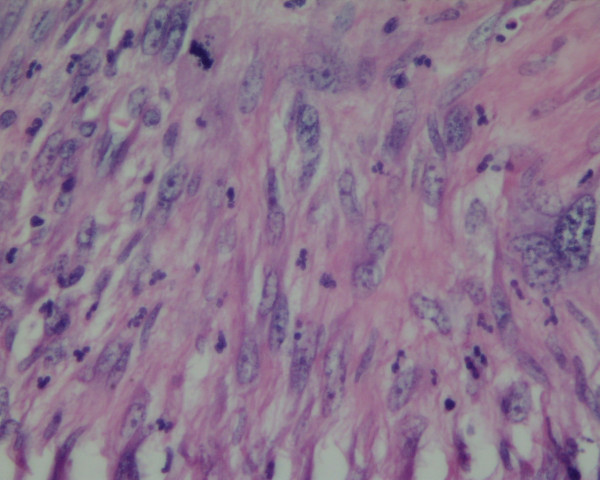
Histopathology image showing spindle to pleomorphic sarcoma with nuclear atypia and mitotic activity.

Microscopically, specimen A showed a mixture of patterns; in some areas, spindle cells with hyalinisation and in other, pleomorphic cells mixed tumour and osteoclast giant cells. Focally, tumour cells had long thin cytoplasmic processes and wavy nuclei. In addition, there was a prominent inflammatory cell infiltrate in the background with foamy histiocytes. Also, the nuclear atypia was marked with a brisk mitotic activity. Immunohistochemistry of the tumour showed smooth muscle actin (SMA) to be focally positive, but Desmin, CD34, S-100, Neurofilament, EMA, CAM 5.2, CD117, CD21 and CD23 were negative. All these appearances were suggestive of a high-grade sarcoma with no discernible line of differentiation; hence it was best classified as grade 3 malignant fibrous histiocytoma. Specimen B showed spindle cell sarcoma with cells showing elongated thin cytoplasmic processes and wavy nuclei. Specimen C showed spindle-pleomorphic sarcoma (Figure 2).

**Figure 3:**
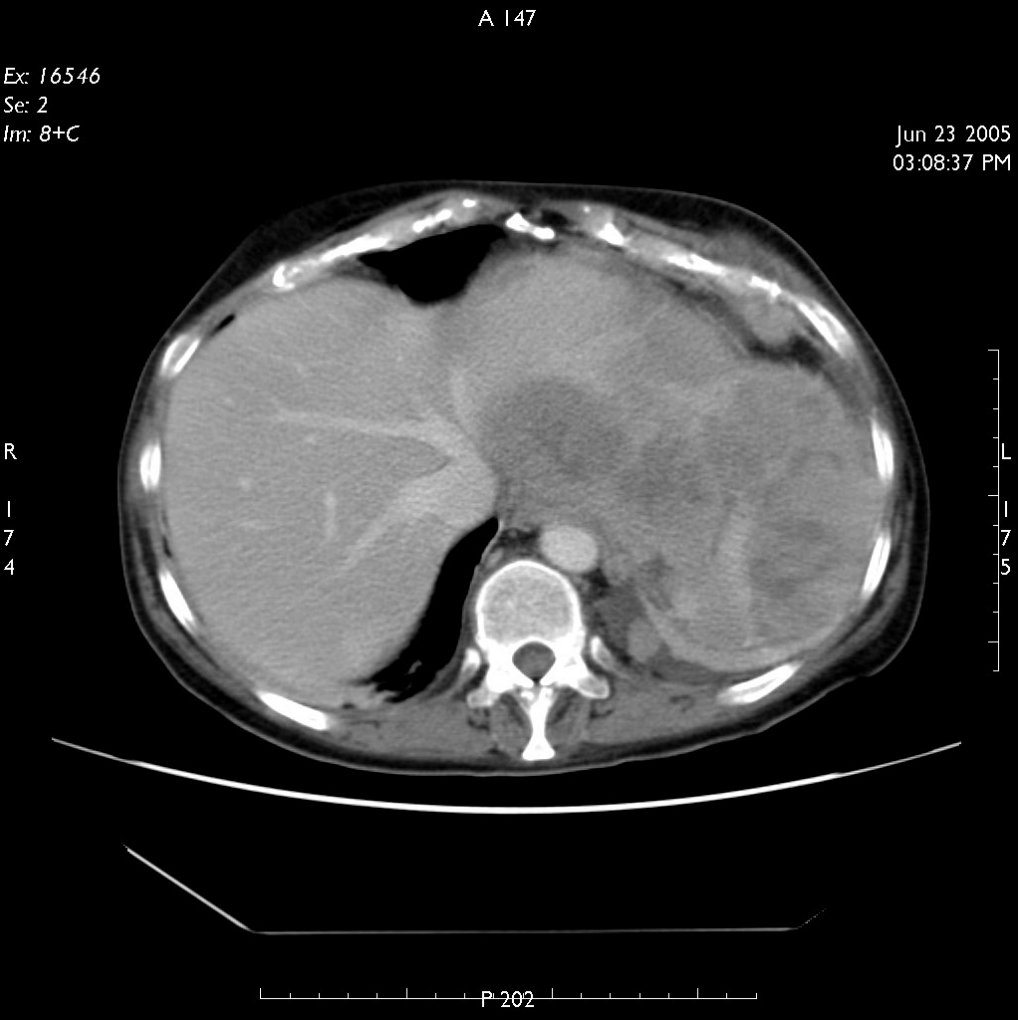
CT scan of abdomen showing local recurrence of the tumour.

**Figure 4:**
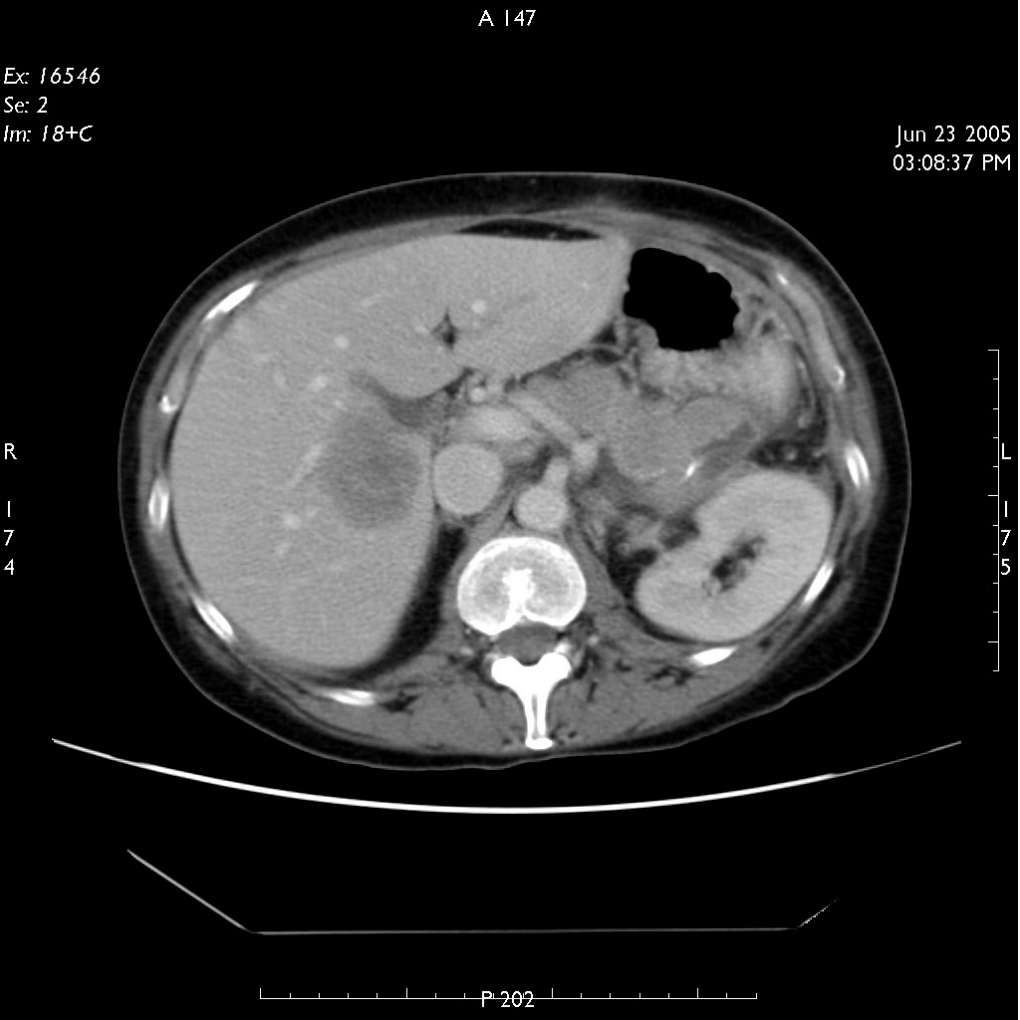
CT scan of abdomen showing liver metastases.

Two months following resection of the tumour, the patient again developed high-grade fever. This was associated with left shoulder pain and weight loss. Investigations showed anaemia and hypercalcaemia. On CT scanning, she was found to have local recurrence of the tumour (figure 3) as well as liver metastases (figure 4). At this stage, patient was referred to the oncology department but unfortunately died 8 weeks later.

## Discussion

Malignant Fibrous Histiocytoma is a sarcoma of mesenchymal origin affecting soft tissues of the body, particularly the extremities and retroperitoneum. Rarely, it may affect intra-peritoneal organs. Its occurrence has been reported in almost all parts of the body including head and neck [2], intracranial [3,4], intra-abdominal organs [5,6] and heart muscle [7]. It is considered as the most common soft tissue sarcoma of the adults [8], but may occur in children as well as infants [9].

The mode of presentation of MFH depends on the primary site of the body affected by the tumour, for example, dyspnoea and arrhythmias can be caused by cardiac lesions. In addition, symptoms of systemic illness caused by the tumour may also be the presenting complaint. Reporting our patient, intra-abdominal MFH presented as pyrexia of unknown origin, caused likely by tumour necrosis and release of inflammatory and pyrogenic factors.

On the basis of its wide range of histological appearances, Enzinger and Weiss subdivided MFH into five sub-types [10,11]:

1. Storiform-pleomorphic

2. myxoid (myxofibrosarcoma)

3. Giant cell (malignant giant cell tumor of soft parts and has the worst prognosis)

4. Inflammatory (xanthosarcoma and malignant xanthogranuloma)

5. Angiomatoid.

Investigations for the diagnosis of MFH include routine haematological, biochemical and radiological tests. A CT scan of the abdomen should be performed early in a patient who presents with abdominal pain and pyrexia of unknown origin but no obvious source of symptoms. This can help identify and localise tumours, if and define extent of growth and presence of metastatic disease. The final diagnosis of MFH is based primarily on the histopathological examination and immunohistochemical reactivity. These diagnostic procedures rely on several criteria, which include the presence of typical spindle and polygonal (strap-like) cells that are filled with an abundant eosinophilic cytoplasm, cells with cross-striations, and, particularly, desmin- and myoglobin-positive immunoreactivity [12,13]. However, the presence of cytoplasmic filaments is not always found in MFH and makes it harder to establish a reliable differential diagnosis.

MFH is an aggressive tumour with a high potential of metastasis to other parts of the body. The Liver is the most commonly involved site of metastatic sarcomas, occurring in 64%–70% of patients [15,16]. The current treatment of choice for primary malignant fibrous histiocytomas is surgical resection [14,15], which involves wide excision of the tumour with an aim for tumour free margins. Recurrence of the tumour is not uncommon even when resection margins are tumour free. Metastasis may present months or years after resection of the primary lesion. Treatment for metastatic disease is surgical where possible; palliative surgery may be carried out if complete resection is not possible. The role of adjuvant radiotherapy and chemotherapy is not clear in the case of retroperitoneal and visceral sarcomas. There are studies that suggest no improvement in overall survival after systemic chemotherapy [16-18]. Some advocate the use of chemoembolization for unresectable metastatic sarcomas, which can provide durable tumour response [19].

## Conclusion

In order to improve survival in patients with MFH, complete resection of the primary tumour as well as isolated peritoneal or hepatic metastases should be attempted where possible. An early multidisciplinary approach is important to improve clinical outcome. Our case report shows that primary intra-abdominal MFH can present in unusual ways including high-grade fever of unknown origin. Clinicians must remember this while establishing differential diagnosis for patients with PUO and abdominal pain.

## Competing interests

The author(s) declare that they have no competing interests.
